# Sigma-1 Receptor Agonist TS-157 Improves Motor Functional Recovery by Promoting Neurite Outgrowth and pERK in Rats with Focal Cerebral Ischemia

**DOI:** 10.3390/molecules26051212

**Published:** 2021-02-24

**Authors:** Jun-Jie Shi, Qi-Hui Jiang, Tian-Ning Zhang, Hao Sun, Wen-Wen Shi, Hendra Gunosewoyo, Fan Yang, Jie Tang, Tao Pang, Li-Fang Yu

**Affiliations:** 1 Shanghai Engineering Research Center of Molecular Therapeutics and New Drug Development, School of Chemistry and Molecular Engineering, East China Normal University, 3663 North Zhongshan Road, Shanghai 200062, China; cpu_jay@163.com (J.-J.S.); cpusunhao@163.com (H.S.); sww_mail@163.com (W.-W.S.); fyang@chem.ecnu.edu.cn (F.Y.); 2 New Drug Screening Center, Jiangsu Center for Pharmacodynamics Research and Evaluation, Institute of Pharmaceutical Sciences, China Pharmaceutical University, Nanjing 210009, China; qihuijiang@163.com (Q.-H.J.); sherrykir@163.com (T.-N.Z.); 3 School of Pharmacy and Biomedical Sciences, Faculty of Health Sciences, Curtin University, Bentley, Perth, WA 6102, Australia; hendra.gunosewoyo@gmail.com; 4 Shanghai Key Laboratory of Green Chemistry and Chemical Process, School of Chemistry and Molecular Engineering, East China Normal University, 3663 North Zhongshan Road, Shanghai 200062, China; jtang@chem.ecnu.edu.cn

**Keywords:** sigma-1 receptor, TS-157, focal cerebral ischemia, neurite outgrowth, pERK

## Abstract

Sigma-1 (σ-1) receptor agonists are considered as potential treatment for stroke. TS-157 is an alkoxyisoxazole-based σ-1 receptor agonist previously discovered in our group. The present study describes TS-157 profile in a battery of tests for cerebral ischemia. Initial evaluation demonstrated the compound’s safety profile and blood–brain barrier permeability, as well as its ability to induce neurite outgrowth in vitro. The neurite outgrowth was shown to be mediated via σ-1 receptor agonism and involves upregulation of ERK phosphorylation (pERK). In particular, TS-157 also significantly accelerated the recovery of motor function in rats with transient middle cerebral artery occlusion (tMCAO). Overall, the results herein support the notion that σ-1 receptor agonists are potential therapeutics for stroke and further animal efficacy studies are warranted.

## 1. Introduction

Sigma-1 (σ-1) receptor is one of the two σ receptors’ subtypes that were discovered by Martin in 1976 [1]. The σ-1 receptor is a chaperone protein that regulates ion channel and signaling molecule activity primarily through its translocation and protein–protein interactions when activated after binding to ligands [2]. As an integral membrane protein, σ-1 receptors are mainly distributed in the central nervous system and notably located in the mitochondria-associated endoplasmic reticulum (ER) and plasma membranes [3,4,5]. In the ER membranes, several cellular responses occur following physiological activation of σ-1 receptor, either by cellular stress or agonist stimulation. These responses include the dissociation from binding immunoglobulin protein (BiP) or inositol-requiring enzyme 1 (IRE1), activation of ER stress responses, dissociation from IP_3_ and Ca^2+^ entry into the mitochondria, and transcriptional regulation of nuclear factor κB (NFκB) [3,6,7,8]. While in the vicinity of plasma membrane, σ-1 receptor translocation impacts different membrane proteins, such as kinases, ion channels, G-protein coupled receptors (GPCR), and trophic factor receptors [9,10,11,12,13]. Consequently, σ-1 receptor is known to be involved in a range of signal transduction pathways, including Ca^2+^ signal pathway at ER, various ion channels, transcription factors’ expression, phospholipase and protein kinase activities, and the Rac1-GTP pathway [13,14,15,16].

The σ-1 receptor-mediated neuromodulation has been shown to hold an important role in brain plasticity, learning and memory processes, mood regulation, and brain preservations. As a consequence, there are numerous literature reports on specific σ-1 agonists and antagonists and how these molecules behave in various cellular models of neuromodulatory systems. Of interest, σ-1 agonists are used in the treatment of epilepsy, depression, ischemia, and neurodegeneration, while σ-1 antagonists are directed toward addiction and pain [5,7,17]. Ischemic diseases such as stroke are among the most common disorders of the central nervous system with unclear underlying mechanisms, although some proposed mechanisms include excitotoxicity, apoptosis, neuro-inflammation, and impaired axon regeneration [18,19,20,21,22,23]. For instance, σ-1 receptor agonist RC-33 was reported to promote the NGF-induced neurite outgrowth of PC12 and that activation of σ-1 receptor could enhance neurite elongation of cerebellar granule neuron cells through TrkB signaling [24,25]. In addition, another σ-1 ligand PRE-084 was found to reduce infarct volume after embolic stroke in rats, improve neurobehavioral function, inhibit pro-inflammatory cytokines, and enhance the expression of anti-inflammatory cytokines [26].

**Figure 1:**
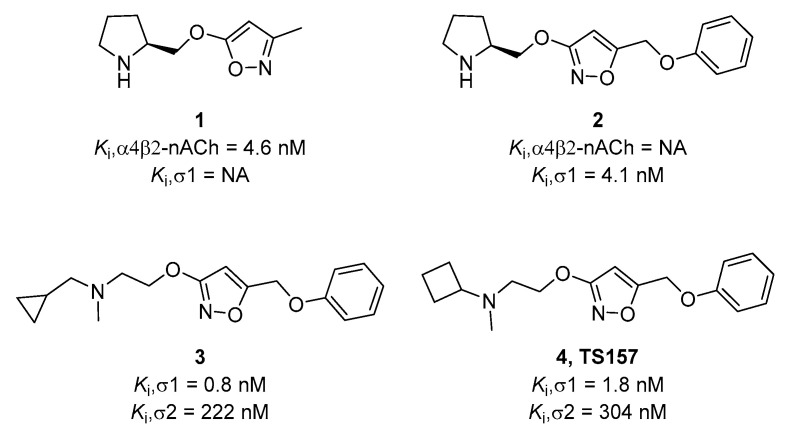
Chemical structures of selected alkoxyisoxazoles.

In previous work, our group designed and synthesized σ-1 receptor ligand **2** containing an alkoxyisoxazole skeleton from the nicotinic receptor ligand **1** by comparing the pharmacophore models [27]. (Figure 1) The tested alkoxyisoxazole showed high selectivity toward σ-1 receptor over σ-2 receptor and a variety of neurotransmitter transporters [27,28,29,30]. Furthermore, σ-1 receptor antagonist **3** and agonist **4** (TS-157) were obtained. The antagonist **3** had a significant analgesic effect in formalin-induced mouse inflammatory model without side effects on motor function [29], while TS-157 induced synaptic elongation in mouse N1E-115 neuronal cells, indicative of its potential to regulate nerve regeneration [30]. In order to further discover the effects of TS-157 in the treatment of cerebral ischemia, we tested the behavioral and biochemical effects upon administration in the rat model with transient middle cerebral artery occlusion (tMCAO) and explored its possible mechanisms.

## 2. Results

### 2.1. In Vitro Non-Cytotoxicity and Membrane Permeability of TS-157

**Figure 2:**
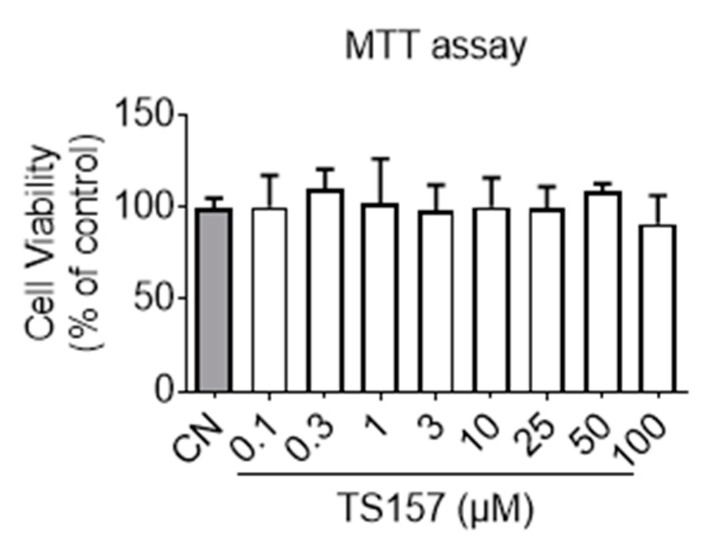
Cell viability of N1E-115 treated with TS-157 at concentrations from 0.1 μM to 100 μM by MTT assay. Data expressed as mean ± SEM.

Before commencing the animal trials, preliminary toxicity profiling and membrane permeation potential of TS-157 were first evaluated in vitro. It can be seen from the results of the MTT (3-(4,5-dimethylthiazol-2-yl)-2,5-diphenyltetrazolium bromide) experiment that TS-157 at a concentration gradient from 0.1 μM to 100 μM had no effect on the viability of the N1E-115 neuronal cells (Figure 2). Next, membrane permeability was evaluated by parallel artificial membrane permeability assay (PAMPA), which tests the passive diffusion of the compound from the donor plate through the lipid membrane into the receptor plate. By measuring the concentration of the compound in the receptor plate, the ability of the compound to penetrate the blood–brain barrier in the body was simulated with good reproducibility, and the results were expressed as Pe (10^−6^ cm/s) [31,32]. In the case of Pe > 4, the compound was considered to have a significant lipid membrane permeability while Pe < 2 indicated poor lipid membrane permeability and Pe = 2–4 reflected a potential lipid membrane permeability. TS-157 exhibited moderate lipid membrane permeability (Pe = (3.15 ± 0.35) × 10^−6^ cm/s) compared to the positive control Atenolol (Pe = (11.3 ± 0.81) × 10^−6^ cm/s) and the negative control Verapamil (Pe = (0.81 ± 0.22) × 10^−6^ cm/s). Furthermore, two calculation models, LogBB [33] and CNS MPO [34], were performed. The latter contains six structural properties, including Clog P, Clog D, MW, TPSA, HBDs, and pKa. Each of these properties was valued between 0 to 1 and, accordingly, the final collective score ranged from 0 to 6, whereby the higher scores were correlated with desirable brain permeability (Table 1).

### 2.2. TS-157 Induces Neurite Outgrowth of N1E-115 Neuronal Cell

A known σ-1 agonist SA-4503 was previously demonstrated to regulate nerve cell plasticity in ischemic stroke animals and contributed to improving the exercise capacity of patients with ischemic stroke [35].

**Figure 3:**
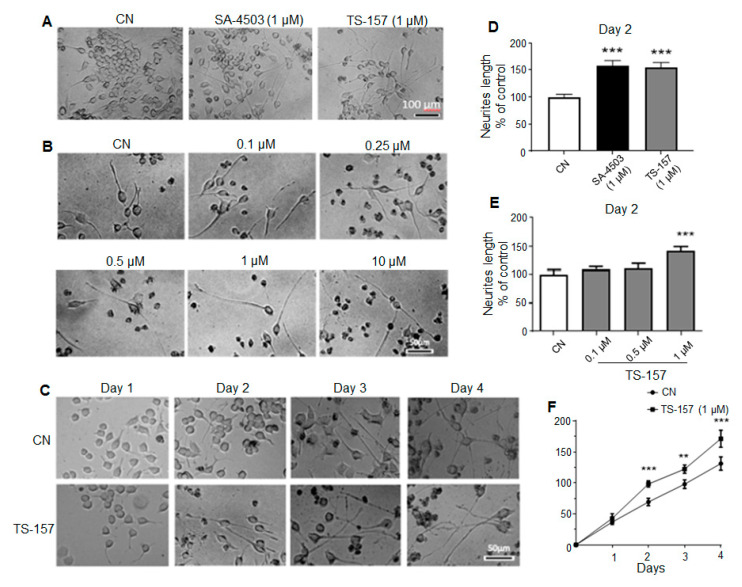
TS-157 concentration- and time-dependently induces neurite outgrowth of N1E-115 neuronal cells. (**A**) Neurite outgrowth induced by SA-4503 and TS-157 at 1 μM; (**B**) neurite outgrowth induced by TS-157 at concentration of 0.1 μM, 0.25 μM, 0.5 μM, 1 μM, and 10 μM; (**C**) neurite outgrowth induced by 1 μM TS-157 in the 1–4 days after administration; (**D**) SA-4503 and TS-157 have similar ability to induce neurite outgrowth at 1 μM; (**E**) TS-157 concentration-dependently induces neurite outgrowth of N1E-115 neuronal cells; (**F**) TS-157 time-dependently induces neurite outgrowth of N1E-115 cells. Data obtained are expressed as mean ± SEM, *n* = 12. Statistically significant differences: ** *p* < 0.01, *** *p* < 0.001 vs. vehicle (one-way or two-way ANOVA followed by Bonferroni test).

Neurite length data on the second day after administration showed that SA-4503 (1 μM) significantly induced the neurite outgrowth of N1E-115 cells similarly to the experimental group treated with 1 μM of TS-157 (Figure 3A,D). As the concentration of TS-157 increased, its effect on inducing N1E-115 neurite growth was also more significant on the second day in a concentration-dependent manner (0.1–10 μM) (Figure 3B,E). In addition, compared to the control group, TS-157 clearly increased the neurite length in the 1–4 days after administration, in which a time-dependent growth of neurites was observed (Figure 3C,F).

### 2.3. TS-157 Induces Neurite Outgrowth through σ-1 Receptor

**Figure 4:**
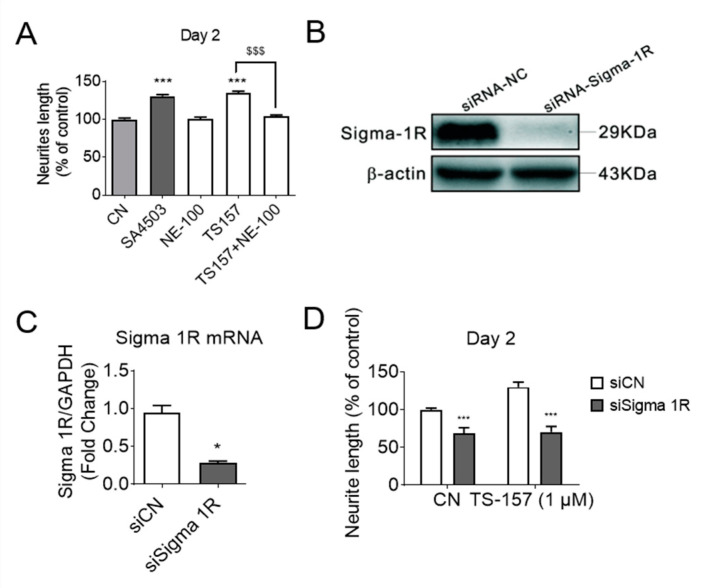
(**A**) The σ-1 antagonist NE-100 (1 μM) attenuates the effect of TS-157 (1 μM) on promoting neurite outgrowth; (**B**,**C**) σ-1 siRNA interferes with receptor expression; (**D**) σ-1 siRNA reverses the effect of TS-157 on extending neurite length. Data obtained are expressed as mean ± SEM, *n* = 12 or 3. Statistically significant differences: * *p* < 0.05, *** *p* < 0.001 vs. vehicle; ^$$$^ *p* < 0.001 vs. ‘TS-157 + NE-100’ (one-way or two-way ANOVA followed by Bonferroni test).

To investigate the relationship between σ-1 receptor modulation and induction of neurite elongation, the σ-1 receptor antagonist NE-100 (1 μM) was used to determine if it could block the neurite elongation induced by TS-157 (1 μM) (Figure 4A). The expression of σ-1 gene was downregulated by σ-1 receptor siRNA, which also significantly reversed the effect of TS-157 on extending the neurite length (Figure 4B–D).

### 2.4. Motor Function Recovery of Long-Term tMCAO Rats by TS-157 Administration

**Figure 5:**
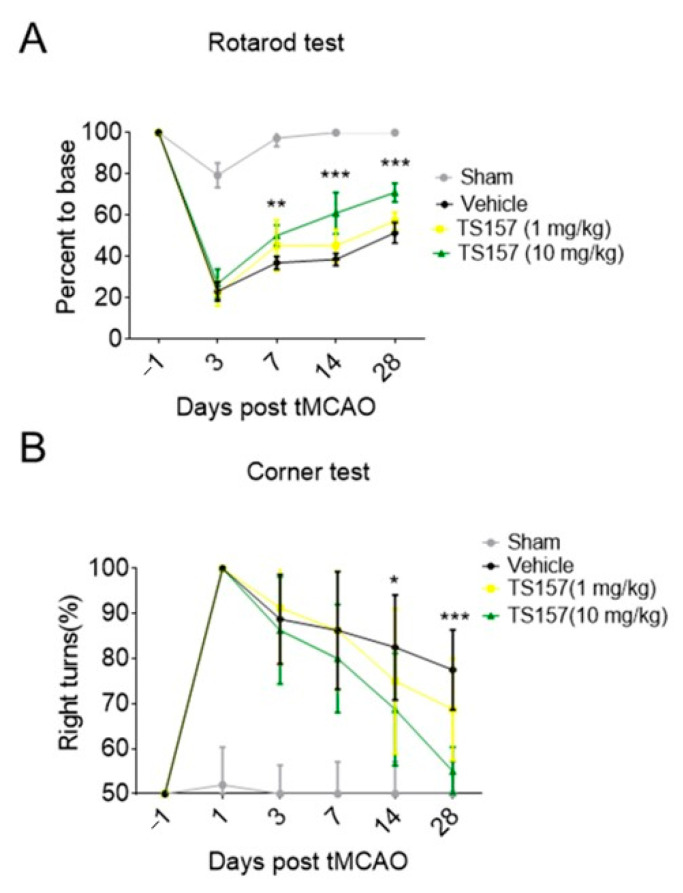
TS-157 significantly improves the exercise ability and movement perception of tMCAO rats. (**A**) Rotarod test for tMCAO rats in sham groups, control group, low-dose group (TS-157, 1 mg/kg), and high-dose group (TS-157, 10 mg/kg); (**B**) corner test for tMCAO rats in above groups. Data obtained from the 8–10 mice per group are expressed as mean ± SEM. Statistically significant differences: * *p* < 0.05, ** *p* < 0.01, *** *p* < 0.001 vs. vehicle (two-way ANOVA followed by Bonferroni test).

The rats in sham group, control group, low-dose group (TS-157, 1 mg/kg), and high-dose group (TS-157, 10 mg/kg) were subjected to a Rotarod test on the first day before surgery and on the third, seventh, 14th, and 28th day after surgery, respectively. The percentage of time that the animal stayed on the rotating rod one day before the operation was used as a basic value for the subsequent corresponding calculations. The percentage of time that the sham group rats stayed on the rotating rod on the third day decreased slightly and then quickly returned to the baseline level. Animals in all the other groups showed a significant decrease in the percentage of time spent on the rotating rod on the third day, followed by a surge thereof in the subsequent 7, 14, and 28 days. Among these three groups, the rats in the high-dose group stayed on the rotating rod for a longer time than the low-dose group, whereby the percentage of time corresponding to both groups was longer than the control group on the 7th, 14th, and 28th day (Figure 5A).

Corner tests were performed on each group of rats on the first day before surgery and on the third, seventh, 14th, and 28th day after surgery. Each rat was tested 10 times and the following formula was used to calculate the probability of turning right: probability of right turn = number of right turns × 100%/total number of turns. The animals in each group had the same probability of turning to the left or right when the operation was not performed. However, the rats in the tMCAO model tended to turn to the right and the probability gradually decreased over time. The probability of turning right in the low-dose group was slightly lower than that of the control group on the 14th and 28th day, while the probability of the high-dose group was significantly lower than the control group on the 14th (*p* < 0.05) and 28th (*p* < 0.001) day (Figure 5B).

### 2.5. No Amelioration for Cerebral Infarct Volume of tMCAO Rats by TS-157

**Figure 6:**
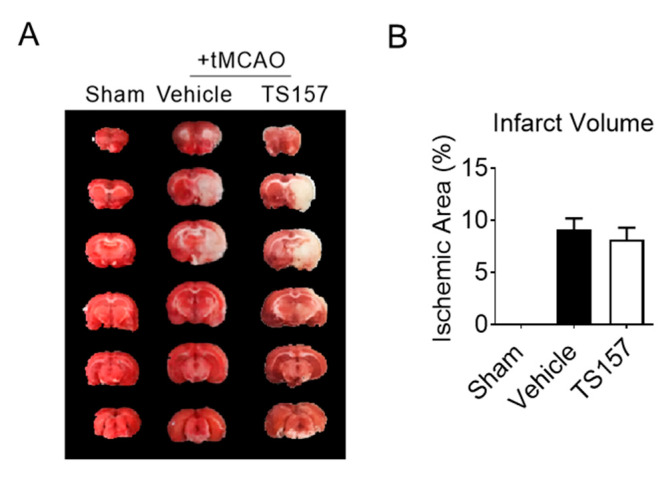
TS-157 has no amelioration effect for cerebral infarct volume. (**A**) Brain tissue of rats in sham group, control group, TS-157 group (10 mg/kg) by TTC staining, and white area represents ischemic volume; (**B**) infarct volume of brain of tMCAO rats.

The tMCAO rats were injected with 10 mg/kg of TS-157 after 24 h of ischemia. Their brains were then removed to be analyzed histologically on the seventh day after the ischemic stroke. The size of the white part in Figure 6 represents the cerebral ischemic volume of rats on the seventh day after the surgery. The volume of the white part of the experimental group was similar to the control group. This, in turn, indicated that TS-157 cannot ameliorate the cerebral ischemic volume of tMCAO rats and it may have no neuroprotective effects on brain injury.

### 2.6. The σ-1 Antagonist Reverses the Recovery of Motor Function in tMCAO Rats

**Figure 7:**
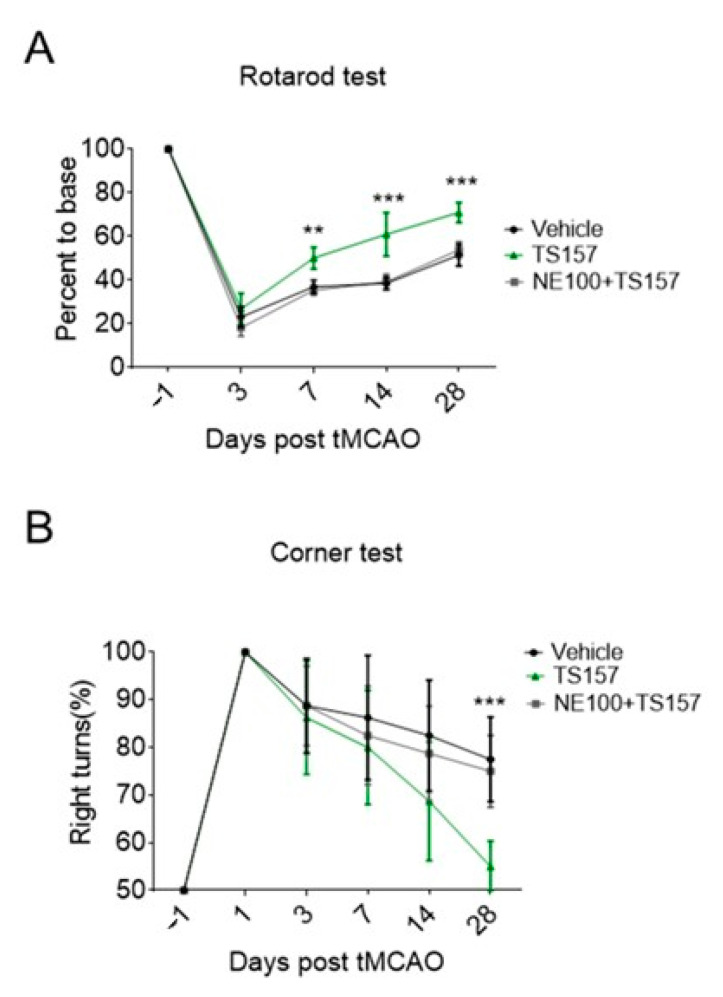
NE-100 reverses the recovery of motor function in tMCAO rats. (**A**) Rotarod test for tMCAO rats in control group, TS-157 group (10 mg/kg), and TS-157 plus NE-100 group (TS-157 10 mg/kg + NE-100 10 mg/kg); (**B**) corner test for tMCAO rats in above groups. Data obtained from the 8–10 mice per group are expressed as mean ± SEM. Statistically significant differences: ** *p* < 0.01, *** *p* < 0.001 ‘NE-100 + TS-157’ vs. ‘TS-157’ (two-way ANOVA followed by Bonferroni test).

In order to determine the mechanism by which TS-157 restores the locomotor function in tMCAO rats and to investigate whether it performs the corresponding function in vivo through the σ-1 receptor, NE-100 as an antagonist was intraperitoneally injected into the rats one hour before TS-157. Both the residence time on the rotating rod and the probability of turning right of rats injected with both NE-100 and TS-157 turned out to be significantly worse compared to TS-157-only group, and the motor function of rats were similar to those of the control group (Figure 7A,B).

### 2.7. ERK Pathway Participates in the Protection of Exercise Capacity of TS-157

**Figure 8:**
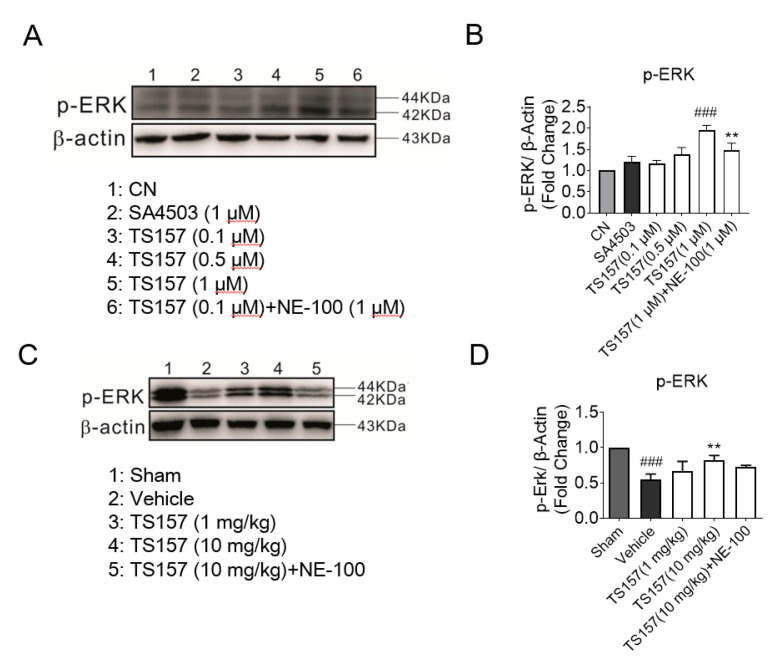
TS-157 can induce upregulation of pERK. (**A**) Western blot for pERK protein of N1E-115 cells with SA4503 (1 μM), TS-157 (0.1 μM, 0.3 μM, 1 μM), and TS-157 (1 μM) plus NE-100 (1 μM); (**B**) pERK/β-Actin ratio of rats in above groups; (**C**) Western blot for brain penumbra protein of rats at 28th day after tMCAO in sham group, control group, TS-157 low-dose group (1 mg/kg), TS-157 high-dose group (10 mg/kg), and TS-157 high-dose plus antagonist group (TS-157, 10 mg/kg + NE-100, 10 mg/kg); (**D**) pERK/β-Actin ratio of rats in above groups. Statistically significant differences: ** *p* < 0.01, ^###^ *p* < 0.001 vs. vehicle (one-way ANOVA followed by Bonferroni test).

The ERK pathway is implicated in many diseases and it mainly regulates the development of the neurons in the central nervous system. ERK is a downstream protein of a variety of growth factors including EGF, NGF, and PDGF, which mediates and amplifies signals in the process of neural signal transmission [36]. For N1E-115 cells cultured with TS-157 (1 μM), the expression level of the phosphorylated ERK (pERK) protein was significantly increased, while SA-4503 showed no significant improvement. Additionally, the effect of TS-157 on upregulating pERK was more pronounced than that of SA-4503 and this effect was inhibited by the antagonist NE-100 (Figure 8A,B). On the 28th day after tMCAO surgery, tissue protein was extracted for Western blot detection from the rat brain in the sham group, control group, TS-157 low-dose group (1 mg/kg), TS-157 high-dose group (10 mg/kg), and TS-157 high-dose plus antagonist group (TS-157, 10 mg/kg + NE-100, 10 mg/kg). Downregulation of pERK protein was seen in the rat brain tissue in the vehicle group compared with that of the sham group (*p* < 0.001). Following administration of TS-157, the expression of pERK protein was increased in a concentration-dependent manner with statistical differences (*p* < 0.01) between the high-dose group (10 mg/kg) and the vehicle group. As expected, these effects were inhibited by the σ-1 antagonist NE-100 (Figure 8C,D).

## 3. Discussion

Stroke is the most common neurological disease that has attracted wide attention because of severe sequelae and younger age of onset. Despite extensive research, there is still no effective treatment for this disease. Stroke has been proven to cause irreversible damage to brain tissue and, although the blood supply gradually recovers later, the damaged neurite is difficult to regrow [37]. Several σ-1 receptor agonists have shown beneficial effects in stroke models, conferring neuroprotection and neurite outgrowth along with other identified mechanistic pathways [24,25,26]. An alkoxyisoxazole-based TS-157 has been demonstrated by our group to be a σ-1 receptor agonist, and this current study explored its potential therapeutic utilities in a stroke model.

At first, the preliminary safety profile, blood–brain barrier permeability, and ability to induce neurite outgrowth in vitro were evaluated. It can be seen from the results of the MTT experiment that TS-157 had no effect on the viability of the N1E-115 neuronal cells, indicating absence of general cytotoxicity. TS-157 exhibited moderate lipid membrane permeability (Pe = (3.15 ± 0.35) × 10^−6^ cm/s) in PAMPA set, which indicated that TS-157 may have the potential to penetrate the blood–brain barrier in vivo. The obtained LogBB value of –0.005 (ideally between 0 and 1) and CNS MPO value of 5.4 (ideally > 4.5) further support the likelihood that TS-157 is able to cross the BBB. TS-157 was shown to increase the neurite length in a time- and concentration-dependent manner. If σ-1 receptor antagonist NE-100 was simultaneously used or the expression of σ-1 gene was downregulated by σ-1 receptor siRNA, the effect of TS-157 on extending the neurite length was significantly reversed, which indicated that TS-157 promoted the neuronal neurite outgrowth through σ-1 receptor.

Next, the corner test and rotarod test were used for locomotion evaluation. The rats with the high-dose TS-157 stayed on the rotating rod for a longer time than the low-dose group and the control group on the different time nodes. The probability of rats turning right in the high-dose group was significantly lower than other groups. These results suggested that TS-157 could restore the exercise ability of the tMCAO rats and accelerate the recovery of their movement perception. In order to further investigate whether TS-157 performs the corresponding function in vivo through the σ-1 receptor, NE-100 as an antagonist was used for evaluation. The results showed that the motor function of rats injected with NE-100 and TS-157 were similar to those of the control group and proved that the effect of TS-157 on restoring the motor function of tMCAO rats was antagonized by NE-100 and that TS-157 acts through the σ-1 receptor. However, the cerebral ischemic volume of rats with TS-157 on the seventh day after the surgery was similar to the control group, which indicated that TS-157 cannot ameliorate the cerebral ischemic volume of tMCAO rats and it may have no neuroprotective effects on brain injury. In some similar studies, different σ-1 agonists showed opposite influence on volume of cerebral ischemia. For example, N, N-dimethyltryptamine was proven reduce infarct size and improve functional recovery following transient focal brain ischemia in rats [38], while another research reported that the treatment of rats subjected to permanent or transient middle cerebral artery occlusion with agonist of the σ-1 receptor enhanced the recovery of lost sensorimotor function without decreasing infarct size [39]. Combining the outcome of that, TS-157 promoted the neurite outgrowth of N1E-115 neuronal cells but could not alleviate brain damage in rats with ischemia. It is proposed that the effective recovery of rat motor function is mainly mediated through neurite growth rather than neuroprotection.

Finally, at the molecular level, we found that ERK was involved in σ-1 agonist inducing neuronal regeneration. ERK is a downstream protein of a variety of growth factors including EGF, NGF, and PDGF, which mediates and amplifies signals in the process of neural signal transmission. In amyotrophic lateral sclerosis mouse models, the downregulation of phosphorylated Akt and ERK1/2 was observed; σ-1 agonist SA4503 could upregulate the levels of phosphorylated Akt and ERK1/2 in a time-dependent manner [40]. In our study, the expression level of the phosphorylated ERK (pERK) protein for N1E-115 cells cultured with TS-157 was significantly increased, which was more pronounced than that of SA-4503 and could be inhibited by NE-100. In tMCAO model, the rats injected with TS-157 reversed the reduction of pERK compared with the vehicle group, and the expression of pERK protein was increased in a concentration-dependent manner. These effects were inhibited by the σ-1 antagonist NE-100, which further proves that the effect of TS-157 on improving tMCAO rat motor function may be related to the σ-1 receptor and involves the ERK pathway.

## 4. Materials and Methods

### 4.1. Preparation of TS-157

The synthesis of TS-157 was accomplished, as reported previously [30]. The purity 98.8% was detected by analytical HPLC, which was carried out on an Agilent 1200 HPLC (Agilent Technologies Inc., Santa Clara, CA, USA) system with a ZORBAX Eclipse XDB-C18 column (Agilent Technologies Inc., Santa Clara, CA, USA), with detection at 220 and 270 nm on a variable wavelength detector G1365D, flow rate = 1.4 mL/min, gradient of 0−100% methanol in water (both containing 0.05 vol% of TFA) in 25 min.

### 4.2. MTT Assay

Cell viability was determined by MTT assay, as reported previously [41]. N1E-115 cells, the mouse neuroblastoma cell lines, were obtained from the American Type Culture Collection (ATCC, Manassas, VA, USA). Briefly, N1E-115 cells cultured in 96-well plates were treated with DMSO or various concentrations of TS-157 (0.1, 0.3, 1, 3, 10, 25, 50, 100 μM) for 48 h in DMEM. Then cells were incubated with MTT (5 mg/mL) for 4 h at 37 °C in humidified atmosphere of 5% CO_2_ and 95% air at 37 °C. The medium with MTT was then removed, DMSO was added to each well, and absorbance was measured at 490 nm in a TECAN (Tecan, Shanghai, China) plate reader.

### 4.3. PAMPA Test

The ability of compounds to cross the cell membrane was predicted and evaluated using PAMPA (Pion Inc., Billerica, MA, USA), as reported previously. A 300 μL filtered secondary stock solution (25 mg/mL) of tested compounds was added to the donor well. The filter membrane was coated with 4 μL of porcine polar brain lipid solution in dodecane (20 mg/mL) and the acceptor well was filled with 150 μL of PBS. The acceptor plate was carefully put on the donor plate to form a sandwich, which was composed of the donor with tested compounds on the bottom, artificial lipid membrane in the middle, and the acceptor on the top. The sandwich was incubated at room temperature for 18 h, and then the donor plate was removed. The concentrations of tested compounds in the acceptor and reference solutions were determined by Safire 2 UV (Tecan, Shanghai, China) plate reader. Every sample was analyzed under three wavelengths in three wells and in three independent runs. *P_e_* was calculated by the following formula:

(1) $$P_{e}=-\frac{V_{dn}V_{ac}}{st(V_{dn}+V_{ac})}\ln\{1-\frac{{\left[drug\right]}_{ac}}{{\left[drug\right]}_{ref}}\}$$

*V_dn_* (μL) is the volume of solution in each well of the donor plate, *V_ac_* (μL) is the volume of solution in each well of the acceptor plate, *[drug]_ac_* is the OD value of the solution in the well of the acceptor plate, *[drug]_ref_* is reference OD value of the solution, *s*(cm^2^) is filter membrane area, *t*(s) is incubation time.

### 4.4. Neurite Outgrowth in N1E-115 Cells

Undifferentiated N1E-115 cells were cultured in DMEM with 100 U mL^−1^ of penicillin, 10% fetal bovine serum, and 100 mg mL-1 of streptomycin and maintained at 37 °C in a humidified incubator supplemented with 5% CO_2_. N1E-115 cells were seeded 1 × 104 cells/mL on poly-l-lysine-coated 96-well plates and grown with DMSO or tested compounds in different concentration (0.1, 0.3, 1 μM) for four days. Every day after incubation, morphometric analysis was performed on digitized images of live cells taken, and images of five fields were taken per well, with about 100 cells per field. The length of neurites was calculated by Image-pro plus (Ipp). All experiments were performed at least five times.

### 4.5. RNA Isolation and Quantitative Real-Time PCR

Total RNA was extracted from the brain cortex by using Trizol reagent (Invitrogen) (Thermo Fisher Scientific, Waltham, MA, USA). Isolated RNA was reverse-transcribed into cDNA by using cDNA synthesis kit (Invitrogen) according to standard protocols. Quantitative PCR (qPCR) was performed at 95 °C for 10 min, 40 cycles of 95 °C for 15 s, and 60 °C for 60 s by using synthetic primers and SYBR Green (Invitrogen) with an IQ5 Detection System (Bio-Rad, Hercules, CA, USA).

### 4.6. Western Blotting

At 24 h after tMCAO, rat brain cortex samples were collected and homogenized with RIPA buffer (Vazyme, Jiangsu, China). The extracted protein was quantified by BCA kit (Thermo Fisher Scientific, Illinois, USA). Western blot was performed, as previously described [42]. Briefly, proteins were separated by SDS-PAGE gels and then transferred onto polyvinylidene difluoride (PVDF) membranes. The membranes were blocked for 2 h with 3% bovine serum albumin (BSA) and then incubated overnight at 4 °C with primary antibodies including σ1 receptor, *p*-ERk, and β-actin. After washing for five times (6 min per wash) with TBST, the membranes were incubated with the secondary antibodies for 1 h at room temperature. The membranes were then washed again and the transferred proteins were visualized with a Bio-Rad ChemiDoc XRS.

### 4.7. The tMCAO in SD Rats

All animals were housed under standard environment and had free access to water and food. All procedures were conducted according to the NIH Guide for the Care and Use of Laboratory Animals. All animal tests and experimental procedures were approved by the Administration Committee of Experimental Animals in Jiangsu Province and the Ethics Committee of China Pharmaceutical University. The healthy male Sprague–Dawley (SD) rats (260–280 g) were randomly selected for transient middle cerebral artery occlusion. Firstly, the rats were treated with anesthesia in 3.5% isoflurane, and then the animals were placed on a heating device to ensure normal body temperature. After the right common carotid artery (CCA), internal carotid artery (ICA), and external carotid artery (ECA) of individual rats were surgically exposed, a monofilament nylon suture with a rounded tip was inserted through the ECA into the ICA and gently pushed about 18 mm to the MCA. After 2 h of cerebral ischemia, the filament was removed to restore blood flow (reperfusion). Sham-operated control rats received the same surgical procedure without insertion of a filament. All rats had free access to food and water. At 24 h after the ischemia, the experimental groups of rats were intraperitoneally injected with 1 mg/kg or 10 mg/kg of TS-157 (dissolved in saline), respectively, while the sham and vehicle groups received saline alone. NE-100 10 mg/kg was given by intraperitoneal injection at 1 h before TS-157 administration.

### 4.8. Rotarod Test

Motor behavior recovery was assessed by the ability to stay on the rod of rats after tMCAO with or without treatment of TS-157. In this experiment, rats were tested seven days before injury, and at 1–28 days post-injury. Animals were placed in an accelerating rotating rod from 4 rpm to 40 rpm during the training period, and finally each rat was kept for 5 min without dropping the rod. The latency to fall off the rotating rod was recorded three times daily until 28 days after brain ischemia. The final data were expressed as the mean value from three trails.

### 4.9. Corner Test

The sensorimotor asymmetry of rats after tMCAO was evaluated by the Corner test. The experimental device consists of two cardboards forming an angle of 30°. When the rats enter it, if both sides of their vibrissae touched corner, rats turned back to face the open side. Normal rats have the same tendency of going left side or right one, while the rats after tMCAO usually turn to the side of the brain injury. The numbers of left and right over 10 trials were recorded.

### 4.10. Measurement of Infarct Size

Rats were euthanized at seven days after tMCAO, and then the brains were collected, dissected on ice, and cut into 2-mm coronal sections. Sections were soaked in 2% TTC (Sigma-Aldrich, Darmstadt, Germany) phosphate buffer for 20 min and placed in a dark area. After the brain was stained, infarct tissues became white, whereas the normal brain tissues stayed red. The ratio percentages of the infarct areas to the total brain areas were calculated by morphometric analysis using Image-pro plus (MEDIA CYBERNETICS, Rockville, MD, USA).

## 5. Conclusions

In the present study, the non-cytotoxicity of TS-157 was first confirmed in vitro, and its lipid membrane permeability was tested through PAMPA to demonstrate its blood–brain barrier penetration potential. TS-157 effectively induced the neurite elongation of N1E-115 neuronal cells in a concentration- and time-dependent manner, reflecting its ability to induce nerve regeneration. In addition, this effect was proven to be facilitated via σ-1 receptor, as demonstrated by the use of σ-1 receptor siRNA and antagonist NE-100. Simultaneously, in the tMCAO rat experiment, TS-157 significantly improved the exercise ability and movement perception of rats with cerebral ischemia reperfusion. However, it did not relieve the symptoms of cerebral ischemia in rats for infarction, suggesting that TS-157 administration only elicits nerve regeneration rather than a total neuroprotection. Finally, antagonist NE-100 antagonized the effect of TS-157 on improving the motor function in rats with ischemia, suggesting that the in vivo efficacy of the latter is mediated through the σ-1 receptor. We also found that TS-157 could restore the downregulation of pERK caused by cerebral ischemia in rats, indicating that ERK is one of the potential pathways involved in this effect. Considering the favorable drug-like properties, pharmacokinetic properties, and blood–brain barrier permeability [28,29] of the alkoxyisoxazole series of σ-1 ligands, TS-157 is worthy of further investigation in stroke or possibly other neurodegenerative diseases.

## Figures and Tables

**Table 1:** Evaluation of membrane permeability of TS-157.

Compound	Tested Pe 10^−6^ cm/s ^a^	Theoretical Pe 10^−6^ cm/s	LogBB ^b^	CNS MPO ^c^
Verapamil	12.3 ± 0.41	16.0	/	/
Atenolol	0.81 ± 0.22	0.8	/	/
TS-157	3.15 ± 0.35		−0.005	5.4

^a^ See Experimental Section. Pe values were determined by PAMPA assay treated compound with 25 μg/mL. Data are expressed as mean ± SEM. ^b^ Log BB values were calculated from the Clark’s equation: Log BB = −0.0148 × PSA + 0.152 × CLogP + 0.139 [33]. ^c^ Last CNS MPO scores were calculated by the tools reported by T.T. Wager et al. [34].

## Data Availability

Any data can be available from authors.

## Sample Availability

Samples of the compounds are available from the authors.

## References

[1] Martin W.R., Eades C.G., Thompson J.A., Huppler R.E., Gilbert P.E. (1976). The effects of morphine- and nalorphine-like drugs in the nondependent and morphine-dependent chronic spinal dog. J. Pharmacol. Exp. Ther..

[2] Schmidt H.R., Zheng S., Gurpinar E., Koehl A., Manglik A., Kruse A.C. (2016). Crystal structure of the human sigma1 receptor. Nature.

[3] Hayashi T., Su T.P. (2007). Sigma-1 receptor chaperones at the ER-mitochondrion interface regulate Ca^(2+)^ signaling and cell survival. Cell.

[4] Alonso G., Phan V., Guillemain I., Saunier M., Legrand A., Anoal M., Maurice T. (2000). ς1 receptor-related neuroactive steroids modulate cocaine-induced reward. Neuroscience.

[5] Su T.P., Hayashi T., Maurice T., Buch S., Ruoho A.E. (2010). The sigma-1 receptor chaperone as an inter-organelle signaling modulator. Trends Pharmacol. Sci..

[6] Mori T., Hayashi T., Hayashi E., Su T.P. (2013). Sigma-1 receptor chaperone at the ER-mitochondrion interface mediates the mitochondrion-ER-nucleus signaling for cellular survival. PLoS ONE.

[7] Meunier J., Hayashi T. (2010). Sigma-1 receptors regulate Bcl-2 expression by reactive oxygen species-dependent transcriptional regulation of nuclear factor kappaB. J. Pharmacol. Exp. Ther..

[8] Hayashi T., Maurice T., Su T.P. (2000). Ca^(2+)^ signaling via sigma(1)-receptors: Novel regulatory mechanism affecting intracellular Ca(2+) concentration. J. Pharmacol. Exp. Ther..

[9] Aydar E., Palmer C.P., Klyachko V.A., Jackson M.B. (2002). The Sigma Receptor as a Ligand-Regulated Auxiliary Potassium Channel Subunit. Neuron.

[10] Kourrich S., Su T.P., Fujimoto M., Bonci A. (2012). The sigma-1 receptor: Roles in neuronal plasticity and disease. Trends Neurosci..

[11] Navarro G., Moreno E., Bonaventura J., Brugarolas M., Farre D., Aguinaga D., Mallol J., Cortes A., Casado V., Lluis C. (2013). Cocaine inhibits dopamine D2 receptor signaling via sigma-1-D2 receptor heteromers. PLoS ONE.

[12] Martina M., Turcotte M.E., Halman S., Bergeron R. (2007). The sigma-1 receptor modulates NMDA receptor synaptic transmission and plasticity via SK channels in rat hippocampus. J. Physiol..

[13] Kourrich S., Hayashi T., Chuang J.Y., Tsai T.S., Su Y.P., Bonci A. (2013). Dynamic interaction between sigma-1 receptor and Kv1.2 shapes neuronal and behavioral responses to cocaine. Cell.

[14] Morin-Surun M.P., Collin T., Denavit-Saubié M., Baulieu E.E., Monnet F.P. (1999). Intracellular sigma-1 receptor modulates phospholipase C and protein kinase C activities in the brainstem. Proc. Natl. Acad. Sci. USA.

[15] Tsai S.Y., Hayashi T., Harvey B.K., Wang Y., Wu W.W., Shen R., Zhang F., Becker Y.K.G., Hoffer J., Su T.P. (2009). Sigma-1 receptors regulate hippocampal dendritic spine formation via a free radical-sensitive mechanism involving Rac1xGTP pathway. Proc. Natl. Acad. Sci. USA.

[16] Natsvlishvili N., Goguadze N., Zhuravliova E., Mikeladze D. (2015). Sigma-1 receptor directly interacts with Rac1-GTPase in the brain mitochondria. BMC Biochem..

[17] Maurice T., Su T.P. (2009). The pharmacology of sigma-1 receptors. Pharmacol. Ther..

[18] Heissa K., Raffaeleb M., Vanellab L., Murabitoc P., Prezzaventob O., Marrazzob A., Aricòb G., Castracania C.C., Barbagallob I., Zappalàa A. (2017). (+)-Pentazocine attenuates SH-SY5Y cell death, oxidative stress and microglial migration induced by conditioned medium from activated microglia. Neurosci. Lett..

[19] Xu K., Tavernarakis N., Driscoll M. (2001). Necrotic celldeath in *C. elegans* requires the function of calreticulin and regulators of Ca^2^^+^release from the endoplasmic reticulum. Neuron.

[20] Degterev A., Hitomi J., Germscheid M., Chen I.L., Korkina O., Teng X., Abbott D.W., Cuny G.D., Yuan C., Wagner G. (2008). Identification of RIP1 kinase as a specific cellular target of necrostatins. Nat. Chem. Biol..

[21] Nour M., Scalzo F., Liebeskind D.S. (2013). Ischemia-Reperfusion Injury in Stroke. Interv. Neurol..

[22] Peters O., Back T., Lindauer U., Busch C., Megow D., Dreier J.P., Dirnagl U. (1998). Increased formation of reactive oxygen species after permanent and reversible middle cerebral artery occlusion in the rat. J. Cereb. Blood Flow Metab..

[23] Rolls A., Shechter R., Schwartz M. (2009). The bright side of the glial scar in CNS repair. Nat. Rev. Neurosci..

[24] Kimura Y., Fujita Y., Shibata K., Mori M., Yamashita T. (2013). Sigma-1 receptor enhances neurite elongation of cerebellar granule neurons via TrkB signaling. PLoS ONE.

[25] Hayashi T., Justinova Z., Hayashi E., Cormaci G., Mori T., Tsai S., Barnes C., Goldberg S.R., Su T.P. (2010). Regulation of σ-1 receptors and endoplasmic reticulum chaperones in the brain of methamphetamine self-administering rats. J. Pharmacol. Exp. Ther..

[26] Katnik C., Garcia A.S., Behensky A.A., Yasny I.E., Shuster A.M., Seredenin S.B., Petrov A.V., Seifu S., Mcaleer J., Willing A.E. (2014). Treatment with afobazole at delayed time points following ischemic stroke improves long-term functional and histological outcomes. Neurobiol. Dis..

[27] Yu L.F., Tuckmantel W., Eaton J.B., Caldarone B., Fedolak A., Hanania T., Brunner D., Lukas R.J., Kozikowski A.P. (2012). Identification of novel α4β2-nicotinic acetylcholine receptor (nAChR) agonists based on an isoxazole ether scaffold that demonstrate antidepressant-like activity. J. Med. Chem..

[28] Yu L.F., Zhang H.K., Gunosewoyo H., Kozikowski A.P. (2012). From α4β2 nicotinic ligands to the discovery of σ1 receptor ligands: Pharmacophore analysis and rational design. ACS Med. Chem. Lett..

[29] Sun H., Shi M., Zhang W., Zheng Y.M., Xu Y.Z., Shi J.J., Liu T., Gunosewoyo H., Pang T., Gao Z.B. (2016). Development of novel alkoxyisoxazoles as sigma-1 receptor antagonists with antinociceptive efficacy. J. Med. Chem..

[30] Sun H., Wang Y.J., Shi W.W., Yang F., Tang J., Pang T., Yu L.F. (2018). Discovery of N-cyclobutylaminoethoxyisoxazole derivatives as novel sigma-1 receptor ligands with neurite outgrowth efficacy in cells. RSC Adv..

[31] Di L., Kerns E.H., Fan K., Mcconnell O., Carter G.T. (2003). High throughput artificial membrane permeability assay for blood-brain barrier. Eur. J. Med. Chem..

[32] Di L., Kerns E.H., Bezar I.F., Petusky S., Huang Y. (2009). Comparison of blood–brain barrier permeability assays: In situ brain perfusion, MDR1-MDCKII and PAMPA-BBB. J. Pharm. Sci..

[33] Clark D.E. (1999). Rapid calculation of polar molecular surface area and its application to the prediction of transport phenomena. 2. Prediction of blood–brain barrier penetration. J. Pharm. Sci..

[34] Wager T.T., Hou X., Verhoest P.R., Villalobos A. (2016). Central Nervous System Multiparameter Optimization Desirability: Application in Drug Discovery. ACS Chem. Neurosci..

[35] Allahtavakoli M., Jarrott B. (2011). Sigma-1 receptor ligand PRE-084 reduced infarct volume, neurological deficits, pro-inflammatory cytokines and enhanced anti-inflammatory cytokines after embolic stroke in rats. Brain Res. Bull..

[36] Huang H., Liu H., Yan R., Hu M. (2017). PI3K/Akt and ERK/MAPK signaling promote different aspects of neuron survival and axonal regrowth following rat facial nerve axotomy. Neurochem Res..

[37] Sun M.S., Jin H., Sun X., Huang S., Zhang F.L., Guo Z.N., Yang Y. (2018). Free radical damage in ischemia-reperfusion injury: An obstacle in acute ischemic stroke after revascularization therapy. Oxid. Med. Cell. Longev..

[38] Nardaia S., Lászlóa M., Szabób A., Alpárc A., Hanicsc J., Zaholac P., Merkelya B., Frecskad E., Nagya Z. (2020). N,N-dimethyltryptamine reduces infarct size and improves functional recovery following transient focal brain ischemia in rats. Exp. Neurol..

[39] Ruscher K., Shamloo M., Rickhag M., Ladunga I., Soriano L., Gisselsson L., Toresson H., Ruslim-Litrus L., Oksenberg D., Urfer R. (2011). The sigma-1 receptor enhances brain plasticity and functional recovery after experimental stroke. Brain.

[40] Onoa Y., Tanakaa H., Takataa M., Nagaharaa Y., Nodaa Y., Tsurumaa K., Shimazawaa M., Hozumib I., Haraa H. (2014). SA4503, a sigma-1 receptor agonist, suppresses motor neuron damage in in vitro and in vivo amyotrophic lateral sclerosis models. Neurosci. Lett..

[41] Chang R.Y., Zhou R., Qi X., Wang J., Wu F., Yang W.L., Zhang W.N., Sun T., Li Y.X., Yu J.Q. (2016). Protective effects of aloin on oxygen and glucose deprivation-induced injury in PC12 cells. Brain Res. Bull..

[42] Xu Y., Xu Y., Wang Y., Wang Y., He L., Jiang Z., Huang Z., Liao H., Li J. (2015). Telmisartan prevention of LPS-induced microglia activation involves M2 microglia polarization via CaMKKβ-dependent AMPK activation. Brain Behav. Immun..

